# Random forest model can predict the prognosis of hospital-acquired *Klebsiella pneumoniae* infection as well as traditional logistic regression model

**DOI:** 10.1371/journal.pone.0278123

**Published:** 2022-11-29

**Authors:** Shuaihua Fan, Jinlan Lin, Sheng Wu, Xiangdong Mu, Jun Guo

**Affiliations:** 1 School of Clinical Medicine, Tsinghua University, Beijing, China; 2 Department of Respiratory and Critical Care Medicine, Beijing Tsinghua Changgung Hospital Affiliated to Tsinghua University, Beijing, China; 3 Department of Disease Control and Nosocomial Infection Control, Beijing Tsinghua Changgung Hospital Affiliated to Tsinghua University, Beijing, China; 4 Department of Emergency, Beijing Tsinghua Changgung Hospital Affiliated to Tsinghua University, Beijing, China; Kaohsuing Medical University Hospital, TAIWAN

## Abstract

**Objective:**

To explore if random forest (RF) model can predict the prognosis of hospital-acquired *Klebsiella pneumoniae* infection as well as traditional logistic regression(LR) model.

**Methods:**

A total of 254 cases of hospital-acquired *Klebsiella pneumoniae* infection in a tertiary hospital in Beijing from January 2016 to December 2020 were retrospectively collected. Appropriate influencing factors were selected by referring to relevant articles from the aspects of basic clinical information and contact history before infection, and divided into a training set and a test set. Both the RF and LR models were trained by the training set, and using testing set to compare these two models.

**Results:**

The prediction accuracy of the LR model was 87.0%, the true positive rate of the LR model was 94.7%; the false negative rate of the LR model was 5.3%; the false positive rate of the LR model was 35%; the true negative rate of the LR model was 65%; the sensitivity of the LR model for the prognosis prediction of hospital-acquired *Klebsiella pneumoniae* infection was 94.7%; and the specificity was 65%. The prediction accuracy of the RF model was 89.6%; the true positive rate of the RF model was 92.1%; the false negative rate of the RF model was 7.9%; the false positive rate of the RF model was 21.4%; the true negative rate of the RF model was 78.6%; the sensitivity of the RF model for the prognosis prediction of hospital-acquired *Klebsiella pneumoniae* infection was 92.1%; and the specificity was 78.6%. ROC curve shows that the area under curve(AUC) of the LR model was 0.91, and that of the RF model was 0.95.

**Conclusion:**

The RF model has higher specificity, sensitivity, and accuracy for the prognostic prediction of hospital-acquired *Klebsiella pneumoniae* infection than the LR model and has greater clinical application prospects.

## Introduction

*Klebsiella pneumoniae* are Gram-negative facultative anaerobic bacteria with thick capsules that colonize human skin, the nasopharynx and the intestines. *Klebsiella pneumoniae* is a common cause of community-acquired infections and hospital-acquired infections [1]. Studies have shown that 7% to 12% of hospital-acquired pneumonia in U.S. intensive care units is due to *Klebsiella pneumoniae* infection [2, 3]. Therefore, the early prediction of hospital-acquired *Klebsiella pneumoniae* infection is of direct significance to the formulation of clinical diagnosis and treatment and is conducive to the formulation of a more reasonable and individualized treatment plan, improving the survival rate of patients and reducing the economic burden on inpatients.

At present, there have been many studies on the prognostic analysis of hospital-acquired *Klebsiella pneumoniae* infection, most often using statistics-based analytical methods, which calculate the chi-square statistic, P value, OR value and 95% confidence interval to obtain highly correlated risk factors. This method is not only a tedious and complex computational process but also cannot intuitively form an evaluation system with direct clinical significance. Recently, with the development of artificial intelligence (AI), people have begun to expect a simpler and more systematic analytical method to solve some problems in medicine. Machine learning(ML) is a sub-classification of AI; it is a powerful tool for analyzing and summarizing complex datasets, and including a variety of algorithms can provide new ideas for disease prediction [4–6]. Several algorithms have been successfully applied to the diagnosis and prediction of diseases and have achieved good predictive performance, including logistic regression models [7], support vector machines [8], decision trees [9], random forests [10], and convolutional neural networks [11]. Compared with traditional statistical methods, machine learning usually has a high sensitivity and specificity, and its calculation process is relatively simple. The results of ML are often shown as predictive models, which are more convenient for clinical use. Among them, the LR model is usually the preferred algorithm for predicting classification problems [12], and RF models are also often used for classification problems. Li et al. [13] collected 246 cases with bacterial bloodstream infection due to combined invasive *Candida* between January 2013 and January 2018. An random forest (RF) model was used to analyze the related risk factors and prognosis. According to the model, weight was an influencing factor in mortality, which helped to identify patients with poor prognoses early and to adjust the treatment plan in a timely manner.

As we known, traditional logistic regression(LR) model has had a good using in the prognostic of prediction on hospital-acquired *Klebsiella pneumoniae* infection, but we wondered if the RF model based on ML can predict the prognosis of hospital-acquired *Klebsiella pneumoniae* infection as good as LR model.

## Methods

### Ethical approval

This study is in line with the Declaration of Helsinki and was approved by the Beijing Tsinghua Changgung Hospital Ethics Committee with approval number 22220-6-01. After Beijing Tsinghua Changgung Hospital Ethics Committee approval, the study was exempted from informed consent.

### Research subjects

This study collected 254 cases of hospital-acquired *Klebsiella pneumoniae* infection from January 2016 to December 2020 in a tertiary hospital in Beijing, China. According to the definition of existing research, the survival group was defined as cases with an outcome of survival within 30 days after infection, and the death group was defined as cases with an outcome of death within 30 days after infection.

The definition of hospital-acquired infection(HAI) [14]: HAI is a combination of hospital/healthcare acquired/associated infection, it refers to infection acquired in hospital by inpatients, including infection acquired during hospitalization and infection detected after discharge assumed to be linked to the hospital stay. This does not include infections that started before or were present at admission.

Inclusion criteria: (1) With the definition of HAI, hospital-acquired *Klebsiella pneumoniae* infection patients should had complete medical records, examination findings and test results and clear prognostic information; (2) According to the drug sensitivity information, the isolated strain was indentified as drug resistance strain. (Both of extended-spectrum β-Lactamase(ESBLs) and carbapenem resistant enterobacteriaceae(CRE) *Klebsiella pneumoniae* infection are obtained in this study).

Exclusion criteria: Patients who do not meet the inclusion criteria.

### Methods used to obtain information on bacteria and drug sensitivity

According to the National Practice Procedures for Clinical Inspection [15], the clinical specimens were isolated and cultured by a VITEK-2 AST-GN 13 susceptibility card (Biomerier, France).

### Research methods

All data were managed using the Numpy and Pandas libraries of the Python programming language. The code was run with Jupyter software (6.3.0), the operations of the LR model and RF model were compared in the Sklearn database, and the performance evaluation of the models was performed using the Scikit-learn package.

### Model training and evaluation

This study was a retrospective study, therefore, some data were missing; the factors with missing values greater than 50% were removed, and the remaining missing values were filled using the k-nearest neighbors (KNN) algorithm. The processed data were divided into a training set and a test set (70% into the training set and 30% into the test set), and each model was trained and evaluated using 5-fold cross validation. Hierarchical k-fold cross-validation was used to keep the results of each fold consistent with the distribution of results in the study population and increase the accuracy of the model. Then, the test set was used to evaluate the performance, and the accuracy, sensitivity, specificity, positive predictive value, negative predictive value and area under curve(AUC) of the model were calculated to compare different algorithms and thresholds.

### Model interpretation

The SHAP value is a game theory method for model interpretation that provides an explanation of the global model structure based on the combination of several local interpretations of each prediction [16]. In this study, we drew a heatmap showing SHAP interaction values, which was used to visually display the influence of different factors in the model on prognosis. Higher values on the heatmap (i.e., brighter squares) indicate greater influence on model predictions, and red and blue are used to indicate risk factors and protective factors.

## Results

### Baseline information

Before data processing, the collected baseline information is presented as follows(Table 1). Median and quartile were used to represent binary variables, while percentage was used to represent continuous variables.

**Table 1:** Baseline information.

	Death(n = 63)	Survival(n = 191)
Age	70(60,81)	63 (51,75)
Gender(Male)	40 (63.5%)	134 (70.2%)
Any resistance at all(Yes)	41 (65.1%)	127 (66.5%)
Hospitalization days	32 (21,42.5)	28 (19,37.5)
ICU admission	35 (55.6%)	114 (59.7%)
Indwelling gastric tube	59 (93.7%)	169 (88.5%)
Indwelling ureter tube	61 (96.8%)	173 (90.6%)
Use ventilator to assist ventilation	51 (81.0%)	99 (51.8%)
History of human albumin infusion within 3 months	50 (79.4%)	115 (60.2%)
History of EPO infusion within 3 months	12 (19.0%)	32 (16.8%)
History of blood transfusion within 3 months	43 (68.3%)	83 (43.5%)
Suffering from lung disease	8 (12.7%)	7 (3.7%)
Suffering from malignant tumor	13 (20.6%)	17 (8.9%)
Suffering from liver disease	11 (17.5%)	16 (8.4%)
Suffering from heart disease	18 (28.6%)	29 (15.2%)
Suffering from neurological disorder	10 (15.9%)	33 (17.3%)
Suffering from kidney disease	5 (7.9%)	12 (6.3%)
Suffering from diabetes	15 (23.8%)	45 (23.6%)
Suffering from high blood pressure	29 (46.0%)	71 (37.2%)
Surgical history within 3 months	26 (41.3%)	115 (60.2%)
History of lumbar puncture within 3 months	5 (7.9%)	17 (8.9%)
History of chest punture within 3 months	10 (15.9%)	24 (12.6%)
History of arterial puncture within 3 months	15 (23.8%)	27 (14.1%)
History of central venipuncture within 3 months	22 (34.9%)	57 (29.8%)
History of trachea cannula within 3 months	23 (36.5%)	47 (24.6%)
History of tracheotomy within 3 months	9 (14.3%)	40 (20.9%)
History of hormone using within 3 months	57 (90.5%)	158 (82.7%)
History of immunosuppressor using within 3 months	3 (4.8%)	17 (8.9%)
History of penicillin using within 3 months	17 (27.0%)	46 (24.1%)
History of third and fourth generation cephalosporin using within 3 months	53 (84.1%)	157 (82.2%)
History of macrolide using within 3 months	19 (30.2%)	49 (25.7%)
History of carbapenem using within 3 months	48 (76.2%)	107 (56.0%)
History of aminoglycoside using within 3 months	7 (11.1%)	34 (17.8%)
History of quinolones using within 3 months	28 (44.4%)	75 (39.3%)
The infection occurs in the lung	30 (47.6%)	74 (38.7%)
The infection occurs in the abdomen	2 (3.2%)	16 (8.4%)
The infection occurs in the urinary tract	13 (20.6%)	57 (29.8%)
The infection occurs in the blood	11 (17.5%)	29 (15.2%)
The infection occurs in other places	5 (7.9%)	12 (6.3%)
The infection occurs in the digestive tract	2 (3.2%)	4 (2.1%)
CRP	72 (40.885,107.25)	50 (21,101.8)
WBC	11.03 (7.495,16.705)	9.44 (6.65,12.62)
PLT	2.88 (2.4,3.375)	3.21 (2.73,3.93)
PCT	135 (67,212)	202 (133,278)
NEU	8.94 (5.785,15.245)	7.4 (4.72,10.82)
LY	0.71(0.42,1.03)	0.93 (0.48,1.41)
HB	89 (77,102)	100 (85,117)
Albumin	30.2 (26.55,32.8)	32.6 (29.2,36.35)
ACHE	2964 (1790.5,3996)	3940 (2867,5329.5)
CRE	12.5 (7.35,19.8)	6.405 (4.465,10.65)
DD	73.2 (40.5,137.3)	59.8 (47.3,80.3)
Kalium	4.13 (3.745,4.61)	4.07 (3.74,4.38)
APACHE II	22 (20,24)	14 (11,17)
SOFA	15 (12,16)	7 (4,9)
Salvage	52 (82.5%)	91 (47.6%)

(Note: There are some abbreviates in this table, the explanations are as follows: CRP refers to C-reactive protein, WBC refers to White blood cells, PLT refers to platelet, PCT refers to Procalcitonin, NEU refers to Neutrophils, LY refers to Lymphocyte, HB refers to hemoglobin, ACHE refers to acetylcholinesterase, CRE refers to creatinine, DD refers to D-dimer.)

### Model evaluation

**Fig 1:**
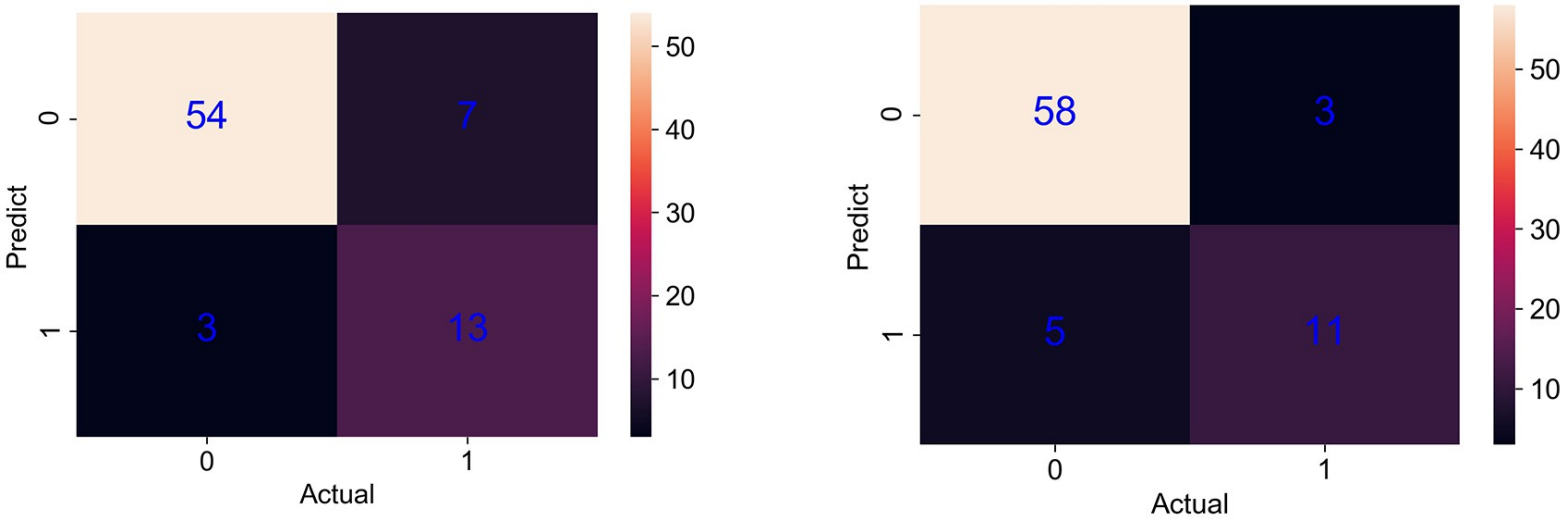
Confusion matrix of the LR model (left) and RF model (right).

The prediction of the models was evaluated by drawing the confusion matrix of the LR model and RF model (Fig 1). There were 77 data points in the test set, and survival outcome was defined as a positive result (represented as 0) and death outcome was defined as a negative result (represented as 1). In the LR model, 54 patients actually survived and were predicted to survive, and the true positive rate was 94.7%. The number of patients who actually survived and were predicted to die was 3; that is, the false negative rate was 5.3%. The number of patients who actually died and were predicted to survive was 7, and the false positive rate was 35%. The number of patients who actually died and were predicted to die was 13, and the true negative rate was 65%. The results showed that the sensitivity and specificity of the LR model for predicting the prognosis of hospital-acquired *Klebsiella pneumonia*e infection were 94.7% and 65%, respectively.

After fitting the data with the RF model, a forest containing 450 trees with a maximum tree depth of 7 was obtained. In the RF model, the number of patients who actually survived and were predicted to survive was 58, and the true positive rate was 92.1%. The number of patients who actually survived and were predicted to die was 5, and the false negative rate was 7.9%. The number of patients who actually died and were predicted to survive was 3, and the false positive rate was 21.4%. The number of patients who actually died and were predicted to die was 11, and the true negative rate was 78.6%. Therefore, the sensitivity and specificity of the RF model for predicting the prognosis of hospital-acquired *Klebsiella pneumoniae* infection were 92.1% and 78.6%, respectively.

**Fig 2:**
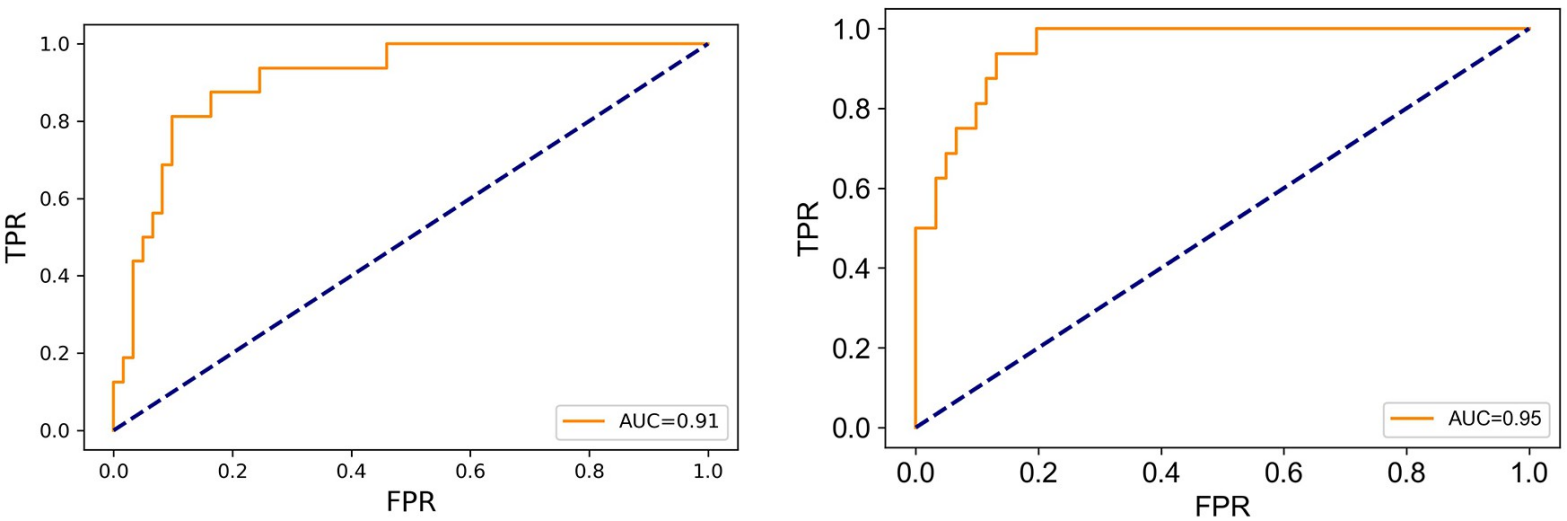
The ROC curves for the LR model (left) and RF model (right).

ROC curves for the LR model and RF model were drawn (Fig 2). The AUC of the LR model was 0.91, and that of the RF model was 0.95.

### SHAP value of the characteristic attributes

**Fig 3:**
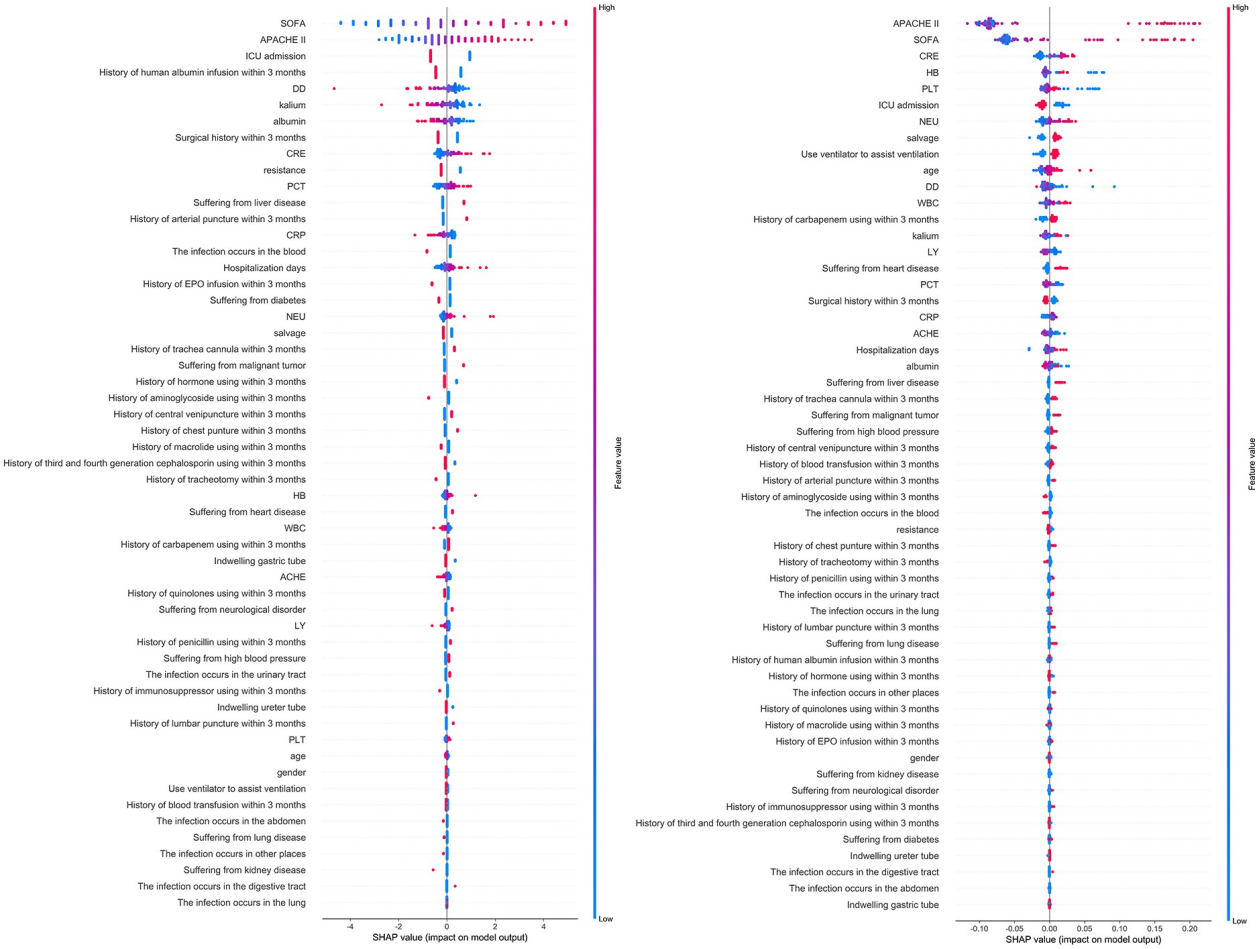
SHAP values of the LR model (left) and RF model (right).

The SHAP package in the Sklearn library was used to draw the interaction of each risk factor in the LR model and RF model (Fig 3). In the expression of SHAP value, red represents death outcome and blue represents survival outcome. Therefore, in the SHAP diagram of the two models, SOFA score and APACHE2 score are the most important factors, and with a higher value, a greater possibility of a death outcome is indicated. In the LR model, the SOFA score was the most influential factor for prognostic prediction, and the top five risk factors were SOFA score, APACHE2 score, ICU hospitalization history within 3 months before infection, history of human albumin infusion within 3 months, and D-dimer level. In the RF model, the APACHE2 score was the most influential factor for prognosis, and the top five risk factors were APACHE2 score, SOFA score, creatinine level, PLT level and HB level.

### The explanation of RF model based on SHAP

**Fig 4:**
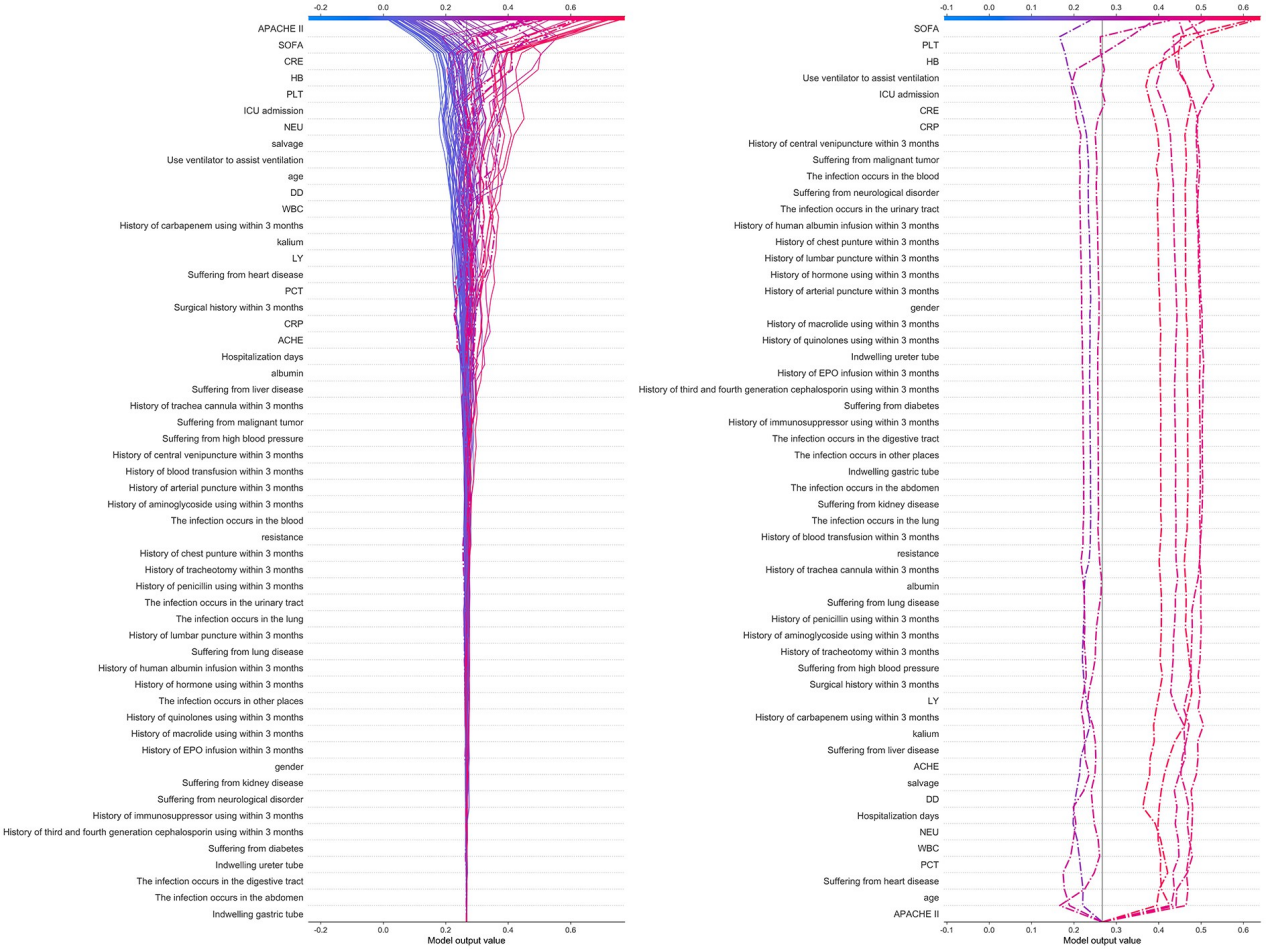
SHAP decision of the RF model (left) and misclassified data (right).

SHAP is used to visually operate the constructed RF model for the prognosis prediction of hospital-acquired *Klebsiella pneumoniae* infection, as shown in Fig 4. The left figure in Fig 4 is the SHAP decision diagram of the RF model, showing how the test set samples can obtain the prediction results through the action of various features in the RF model. Red represents the positive contribution to the survival outcome, while blue represents the negative contribution. The farther the deviation from the center line, the higher the SHAP value of the feature, and the greater influence on the outcome. It is not difficult to find that the features with high importance, such as APACHE II and SOFA scores, turn the decision curve to another way, and have great influence on the generation of the prognostic outcome of each samples in test set, while the underlying features have little effect on each samples.

In addition, according to the confusion matrix, there were 8 samples in the test set were misclassified, and the desicion paths of these 8 samples were separately shown in the right figure in Fig 4. The red line shows patients with survival outcomes misclassified as death, and the blue line shows patients with death outcomes misclassified as survival.

**Fig 5:**
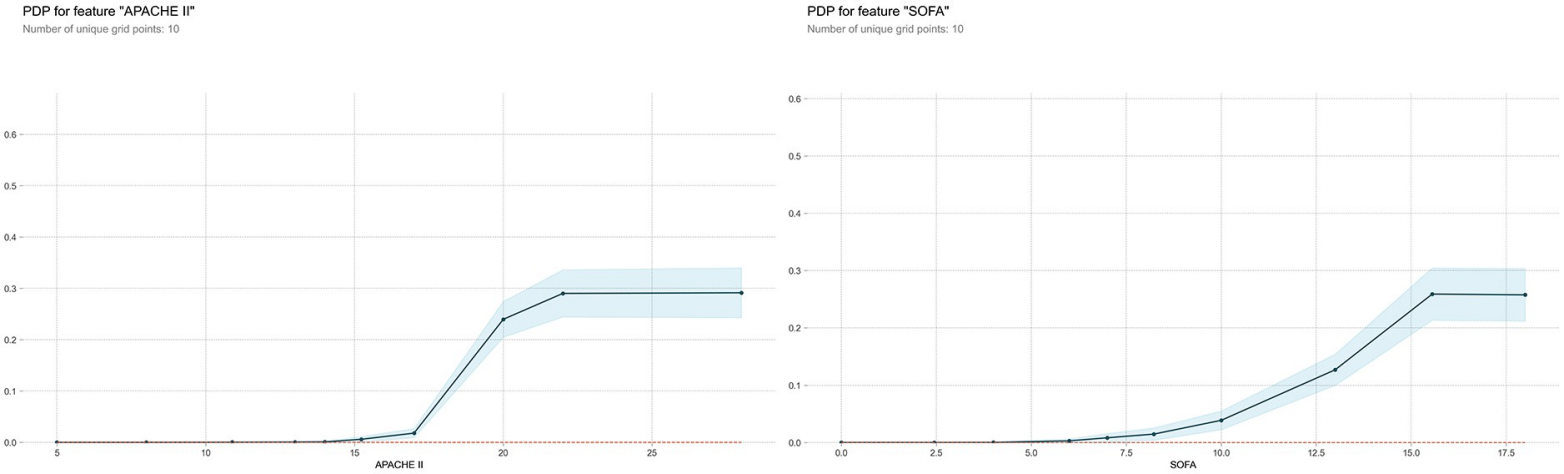
PDP for feature APACHE II (left) and SOFA (right).

In addition, base on Partial Dependence Plot analysis(PDP), the influence curves of the two features with the highest impact are drawn, as shown in Fig 5.

## Discussion

With the development of AI, more cross-disciplines are emerging. Machine learning is a branch of AI, defined by Arthur Samuel as "the field that gives computers the ability to learn that is not acquired through explicit programming". Simply put, machine learning is about feeding data to machines that mimic the patterns of the human brain, looking for patterns and making predictions about the future. At present, machine learning has been gradually applied in the medical field and has achieved good results [17, 18], providing ideas for more accurate and convenient disease prediction and classified diagnosis and treatment.

Karthikeyan et al. [19] used machine learning (neural network, logistic regression, XGBoost, random forest, SVM and decision tree) to integrate clinical information to predict the prognosis of COVID-19 patients and obtained a prognostic model with 96% accuracy. The models were evaluated as early as 16 days before the results and were able to predict outcomes with 90% accuracy. Ma et al. [20] used machine learning (random forest and XGboost) to construct a prognostic prediction model for COVID-19 patients admitted to hospitals in Wuhan from January 15 to March 15 in 2020, and the results showed that the ROC curve and AUC of the models were superior to those of traditional assessment tools. Further data on LDH, CRP, and age can be used to identify patients with severe COVID-19 at admission.

This study shows that the predictive accuracy of the LR model for hospital-acquired *Klebsiella pneumonia*e infection is 87.0%, and the predictive accuracy of the RF model is 89.6%. The RF model has a larger advantage in accuracy; the RF model more easily predicts the existence of a survival outcome in patients with hospital-acquired *Klebsiella pneumonia*e infection, and the recall rate is higher. The sensitivity of the LR model in the prognostic prediction of patients with hospital-acquired *Klebsiella pneumonia*e infection is 94.7%, and the specificity is 65%. The sensitivity of the LR model is higher, but the specificity is poor. It tends to predict an outcome of survival and has a greater probability of predicting survival for patients who have an outcome of death or disease. In clinical application, it is likely to make errors, and there would be a risk of delayed treatment. The sensitivity and specificity of the RF model for the prognostic prediction of patients with hospital-acquired *Klebsiella pneumonia*e infection were 92.1% and 78.6%, respectively. The sensitivity and specificity of the RF model were slightly higher than those of the LR model, and the RF model had obvious advantages over the LR model. It is not easy to miss patients with serious disease, and it is more helpful in providing timely and correct diagnosis and treatment.

In addition, this study instantiates the assignment of each characteristic attribute in the two models, which is represented by the SHAP attribution value. The LR model and RF model both rank the SOFA score and APACHE2 score in the first two values, and many studies have shown that the SOFA score and APACHE2 score have great predictive value for the clinical prognosis of infectious diseases [21, 22].

However, there are some limitations to our study. First, imaging data, such as chest CT and chest X-ray, were missing from the dataset of this study. However, in practical clinical applications, clinicians will not only rely on the information provided by the model for medical treatment but also make comprehensive evaluations based on imaging information to formulate diagnosis and treatment plans. Second, there are many factors affecting the prognosis of patients that cannot be assessed, such as differences in medical resources. The model data obtained in this study can only be used to assist clinical judgment and cannot be used to directly predict clinical outcome. As more data are collected, separate algorithms that are more sophisticated and can cover more uncontrollable factors may be developed in the future. This study is a single-center study with a medium sample size, and the training of the model need amount of data, therefore the model in this study may not be optimal, so there may be some degree of bias in the conclusion. In the future, the sample size may be gradually expanded to continue to optimize the model. When the sample size is large enough, the prediction rate of the model will gradually improved. In the future, it may be possible to package the optimized model into an application so that it can be used at any time, and the model can be optimized by constantly adding new data to truly achieve accurate and early prediction of the prognosis of hospital-acquired *Klebsiella pneumonia*e infection.

## Conclusions

The optimized RF model and LR model both have a good degree of fit to this dataset and can obtain a good area under the curve (both greater than 0.8). However, the RF model has higher specificity, sensitivity, and accuracy for the prognostic prediction of hospital-acquired *Klebsiella pneumoniae* infection than the LR model, RF model has better interpretability and can intuitively display the influence of various factors on the outcome, thus RF model may have greater clinical application prospects.
